# Animal Model for Prodromal Parkinson’s Disease

**DOI:** 10.3390/ijms21061961

**Published:** 2020-03-13

**Authors:** Tomoyuki Taguchi, Masashi Ikuno, Hodaka Yamakado, Ryosuke Takahashi

**Affiliations:** Department of Neurology, Kyoto University Graduate School of Medicine, Kyoto 606-8507, Japan

**Keywords:** Parkinson’s disease, prodromal, animal model, alpha synuclein

## Abstract

Parkinson’s disease (PD) is characterized by the loss of dopaminergic neurons in the substantia nigra and subsequent motor symptoms, but various non-motor symptoms (NMS) often precede motor symptoms. Recently, NMS have attracted much attention as a clue for identifying patients in a prodromal stage of PD, which is an excellent point at which to administer disease-modifying therapies (DMTs). These prodromal symptoms include olfactory loss, constipation, and sleep disorders, especially rapid eye movement sleep behavior disorder (RBD), all of which are also important for elucidating the mechanisms of the initiation and progression of the disease. For the development of DMTs, an animal model that reproduces the prodromal stage of PD is also needed. There have been various mammalian models reported, including toxin-based, genetic, and alpha synuclein propagation models. In this article, we review the animal models that exhibit NMS as prodromal symptoms and also discuss an appropriate prodromal model and its importance for the development of DMT of PD.

## 1. Introduction

Parkinson’s disease (PD) is one of the most common neurodegenerative disorders. It is characterized by motor symptoms such as bradykinesia, rigidity, tremor, and postural instability, but it is also well known that a lot of non-motor symptoms often precede motor symptoms. The current diagnostic criteria for PD are based on motor symptoms [1], but the number of dopaminergic (DA) neurons in the substantia nigra pars compacta (SNc) has already been reduced by about half [2] when patients are diagnosed with PD. At present, only symptomatic therapies are available for PD, so both the establishment of early diagnostic methods and development of disease-modifying therapies (DMTs) that prevent the onset or slow progression of the disease are urgently needed. 

Animal models are essential in a preclinical study for drug development. A lot of models have been created mainly to reproduce DA cell loss and motor symptoms after the onset. However, models for the prodromal stage when the DA cell loss is minimal are also necessary for the development of DMTs in PD, and several models have recently been developed for this purpose [3].

**Figure 1:**
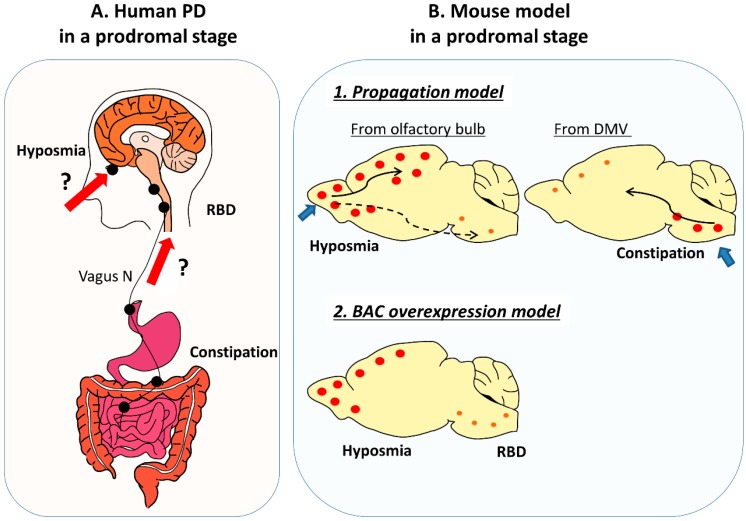
Human Parkinson’s disease (PD) and mouse model in a prodromal stage. (**A**) In human PD, Lewy pathology is already present in a prodromal stage in extra central nervous system (CNS) tissues including GI tract and autonomic nerves, and in the lower brainstem and/or olfactory bulb. As a prodromal symptom hyposmia, constipation, and rapid eye movement sleep behavior disorder (RBD) appear in a chronologically close period, suggestive of the multifocal initiation of PD. Black dots indicate Lewy pathology. Bold red arrows and question marks indicates hypothesized propagation pathway of Lewy pathology in human. (**B**) In a propagation model, α-syn aggregates start from the olfactory bulb or the GI tract/ dorsal nucleus of the vagus nerve (DMV), and hyposmia and GI dysfunction appear as its corresponding symptoms in a prodromal stage. In contrast, in a α-syn BAC transgenic model that mildly overexpresses α-syn in its native expression manner, both of hyposmia and RBD are observed with multifocal accumulation of phosphorylated α-syn. Orange dots indicate accumulateon of phosphorylated α-syn and bold blue arrows indicate entry zone of injected α-syn. Thin solid arrows shows main propagateon pathway of α-syn and dotted arrows indicate minor propagateon pathway in animal experiments.

Among the many prodromal symptoms, hyposmia, constipation, and rapid eye movement (REM) sleep behavior disorder (RBD) are particularly important. The positive likelihood ratios for the future development of PD in individuals with hyposmia, constipation, and polysomnography-confirmed RBD are 6.4, 2.5, and 130, respectively [4]. Furthermore, these prodromal symptoms often appear in combination before the appearance of motor symptoms and attract attention as a clue for determining the initiation process of the PD pathology. The Braak hypothesis states that pathological alpha synuclein (α-syn) propagates from the olfactory bulb and/or the lower brainstem, especially from the dorsal nucleus of the vagus nerve (DMV) [5]. The olfactory bulb and DMV have contact with the outside of the body through the olfactory nerve and gastrointestinal (GI) tract, respectively, suggesting the influence of peripheral factors on the initiation of PD. As for RBD, the nuclei in the ventromedial medulla and locus subcoerules located in the lower brainstem are thought to be the responsible regions [6] (Figure 1). Although the olfactory bulb and these lower-brainstem nuclei are spatially separated, hyposmia and constipation/RBD reportedly appear in a chronologically close period [7], suggesting the multifocal initiation of PD rather than the simultaneous propagation from the periphery. 

At present, mammalian models for prodromal PD can be roughly classified into three categories: toxin-based, genetic, and α-syn propagation models. In this paper, we will focus on olfactory disturbance (hyposmia), constipation, and RBD as important symptoms in the prodromal stage of PD and introduce representative animal models for prodromal symptoms. These models are expected not only to help elucidate the pathomechanisms underlying the initiation and progression of PD but to play important roles as preclinical models for DMT in PD.

### 1.1. Toxin-Based Models

PD is a multifactorial disease caused by the sum of the effects of multiple genetic and environmental factors. Environmental factors include intestinal flora, inflammation, viruses, and toxicants. For example, rotenone, a kind of pesticide, causes DA cell death through mitochondrial toxicity [8,9]. 1-Methyl-4-phenyl-1,2,3,6-tetrahydropyridine (MPTP) and 6-hydroxydopamine (6-OHDA) also act as neurotoxins against DA neurons [10,11]. Toxin-based animal models have greatly contributed to the development of symptomatic treatments, mainly for motor symptoms. Furthermore, these toxins can reproduce the motor complication of anti-Parkinsonian drugs, such as l-dopa-induced dyskinesia, and is also used to check the efficacy of therapies against motor complications in the advanced stage of PD [12]. These models have advantages in that the phenotype is clear, the time required for the experiment is short, and they can be applied to various animal species (Table 1). However, toxin exposure to the experimenter must be avoided, and criticisms that these kinds or concentrations of toxins do not exist in the natural environment have been levied. In addition, robust motor phenotypes can interfere with the analysis of non-motor phenotypes. Nonetheless, some toxin-based models also show prodromal symptoms and will continue to be useful models in the future.

### 1.2. Genetic and α-syn Propagation Models

One of the most important genetic factors in PD is *α-synuclein* (*SNCA*), which is the causative gene of familial PD. *SNCA* encodes α-syn, the main constituent of Lewy bodies (LBs), which are the important pathological feature of PD [13]. Point mutations, such as A53T and A30P, as well as duplication and triplication of *SNCA,* also cause PD [14,15,16]. This means that quantitative and qualitative changes in α-syn can trigger PD. Furthermore, a genome-wide association study (GWAS) in PD showed that the highest risk SNP was localized in *SNCA* [17,18]. 

With this background, a number of α-syn transgenic animal models have been created, contributing to the elucidation of the pathology and treatment for PD [19,20,21,22,23,24,25,26,27]. The α-syn transgenic model can reproduce α-syn aggregation similar to human PD and show slowly progressive changes (Table 1). However, the phenotypes essentially depend on the potent exogenous promoters used to overexpress α-syn and do not necessarily reflect the spatial and temporal distribution of human PD pathologies. In addition, it usually takes time and is sometimes difficult to obtain the desirable phenotypes compared with other methods. 

Other genetic models are also created by overexpression or deletion of genes for familial PD. Genes for familial PD with Lewy bodies and age-dependent progression with non-motor phenotypes are especially important to reproduce the natural course of idiopathic PD. 

Recently, the propagation hypothesis that α-syn can be transmitted by its prion-like property to form a pathological condition of PD has attracted attention. In clinical trials of fetal DA cell transplantation in PD, aggregation of α-syn was observed in grafts more than 10 years after transplantation [28,29]. In mouse experiments, injected α-syn fibrils have been shown to spread throughout the brain [30]. The propagation model created by exogenously administered α-syn fibrils is still in development, but it is relatively easy to obtain a phenotype, and the time required for the experiment is shorter than with the transgenic model (Table 1). It is also a great advantage that this model can be applied to non-human primates and other animals as well. However, even if the propagation hypothesis is true, questions remain concerning where and how the initial α-syn aggregates are formed, and it should be noted that this model is artificial in nature. In addition, the method of preparing and injecting fibrils differs among facilities, which impacts the degree of α-syn propagation and cell loss in this model [31,32,33].

## 2. Prodromal Symptoms and Relevant Animal Models

### 2.1. Prodromal Symptoms of PD Patients

Prodromal symptoms are important as clinical biomarkers to identify patients in a premotor stage, especially when the fluid and imaging biomarkers are lacking. They include hyposmia, sleep abnormalities, autonomic dysfunction, and psychiatric problems, and some of the prodromal symptoms such as anxiety/depression, cognitive decline, and somnolence are much more frequent and become problematic in an advanced stage. These non-motor symptoms should be weighted as prodromal biomarkers in terms of the prevalence in PD patients, diagnostic strength to predict future development of PD, lead time to the diagnosis of PD, and feasibility of assessment and screening [34]. The highest predictive value for the future development of PD is observed in polysomnography-confirmed RBD with a positive likelihood of 130, but their prevalence in the general population is very low (high specificity and low sensitivity) and is not suitable for screening. In contrast, the predictive value of constipation is moderate with a positive likelihood of 2.5, but the prevalence in the general population is very high (high sensitivity and low specificity). Hyposmia has moderately high predictive value with a positive likelihood of 6.4, and its prevalence in the general population is moderate [4].

The pattern and sequence of these prodromal symptoms may provide important information on the initiation and mode of progression of PD, and Braak’s staging would help our understandings [5,35]. In Braak staging, lesions in Stage 1–2 is thought to be responsible for prodromal symptoms. Stage 1 includes the DMV that innervates the GI tract and the olfactory bulb, which may correspond to constipation and olfactory dysfunction, respectively. Constipation is one of the main and most frequent autonomic dysfunctions of PD, and a case–control study showed that constipation can occur more than 20 years before the onset of motor symptoms [36]. In the pathological analysis, α-syn was found to be accumulated in the DMV and enteric nervous system [5,37]. In the GI tract, α-syn pathology is stronger in the rostral part than that in the caudal part [38,39]. Olfactory disturbance has been shown to be associated with not only PD but also many neurodegenerative diseases. It is observed in more than 90% of sporadic PD patients and appears at least several years before the onset of motor symptoms [40]. In accordance with this clinical observation, α-syn aggregates are observed in the olfactory tract, even in early-stage PD patients [5]. The number of tyrosine hydroxylase (TH)-positive cells in the olfactory bulb is markedly increased, and it has been speculated that the inhibition of olfactory transmission by DA may be responsible for the olfactory disturbance in PD [41]. Stage 2 includes the raphe nuclei, gigantocellular reticular nucleus of the medulla oblongata, and the locus coeruleus in the pontine tegmentum [5,35]. These regions can be related to sleep disorders, depression, or anxiety. Depression and anxiety are observed in 30–45% and 25–49% of PD patients, respectively, in all PD stages including the prodromal stage [42]. Sleep disorders are one of the most frequent symptoms in PD and include daytime sleepiness, insomnia, RBD, and restless leg syndrome [43]. Autopsy brains of idiopathic RBD patients have been reported to show Lewy pathology in the brainstem and neurodegeneration in the SNc [44,45]. In autopsy brains of PD patients who developed PD and dementia with Lewy bodies (DLB) preceded by idiopathic RBD, LBs were identified in the pedunculopontine tegmental nucleus (PPN), locus coeruleus/subcoeruleus complex, and gigantocellular reticular nucleus in medulla oblongata [46]. These regions are considered part of the neural circuit controlling atonia during REM sleep in animal experiments [6,47,48] and thus responsible for RBD. In this way the pathological progression of PD largely follows Braak’s staging. However, it should be also noted that the Braak stage does not always correlate with the severity of neuronal loss and associated clinical symptoms [49]. 

In this section we will focus on the hyposmia, constipation, and RBD, considering their importance as prodromal symptoms of PD from the viewpoints of the prevalence in PD patients, diagnostic strength to predict future development of PD, and feasibility of assessment in animals.

### 2.2. Prodromal Symptoms of Toxin-Based Models

In the toxin-based model, neuronal death of SNc appears several days or several weeks after toxin administration [8,9,10,11,12]. Most of toxin-based models do not show LB that matches the disease state, and the site of injury is limited. Therefore, it is difficult to reproduce the chronic progression of the disease along with the Braak stage. However, it is interesting to see how non-motor symptoms can be reproduced by the functional abnormalities in these toxin-based models.

#### 2.2.1. Olfactory Dysfunction in Toxin-Based Models

The intranasal administration of MPTP-reproduced transient olfactory disturbance in rats [50]. Olfactory disturbance was also observed in rats with rotenone administered to the SNc, and DA produced by periglomerular neurons and D2 receptors in the olfactory bulb was thought to be important for the olfactory discrimination ability according to DA agonist/antagonist administration experiments in this model [51].

#### 2.2.2. Constipation in Toxin-Based Models

MPTP exposure in mice has been shown to reduce the number of intestinal dopamine neurons and cause GI dysfunction [52]. However, in this model, intestinal motility was actually increased, and an electrophysiological study suggested the impaired neural-mediated relaxation of the colon. A decreased number of TH cells in the myenteric plexus of the intestinal tract has also been reported in an MPTP-treated primate model [53]. The chronic administration of low-dose rotenone to rats caused α-syn aggregation mainly in the myenteric ganglia of the small intestine, and the number of enteric neurons and GI motility were decreased 6 months after administration [54]. In a rat model with subcutaneous rotenone injection, a longer gastric-emptying time and a transient decrease in stool frequency were observed, but there was no α-syn pathology [55].

#### 2.2.3. RBD in Toxin-Based Models

Increased muscle tone during REM sleep, which is suggestive of an RBD-like phenotype, was reported in a 6-OHDA-treated rat model [56]. In this model, loss of DA neurons in the SNc was considered to be responsible for RBD-like phenotype, as there were no obvious pathological changes in the RBD-related regions, including the sublaterodorsal nucleus, which is equivalent to the locus subcoeruleus in humans; in contrast, a reduction by over 95% in the number of TH-positive neurons was noted in the SNc. In rhesus monkeys and marmosets treated with MPTP, the muscle tone was increased during REM sleep [57,58]. All of the above-mentioned toxin-based models have already shown motor symptoms accompanied by robust DA neuronal death, so these models may actually reflect sleep disorders after the onset of motor symptoms in PD.

### 2.3. Prodromal Symptoms of Genetic Models

#### 2.3.1. α-syn Genetic Models

In α-syn genetic models, it is important to consider that the phenotypes completely depend on promoters. For example, α-syn can be overexpressed in a wide range of neurons by the prion promoter, but it may cause toxicity in undesired regions such as spinal cords. When the TH promoter is used, α-syn can be overexpressed only in PD-vulnerable catecholaminergic neurons, but the effect on other types of neurons such as cholinergic neurons cannot be evaluated. From this viewpoint, transgenic models with endogenous promoters of α-syn using bacterial artificial chromosome (BAC) and P1-derived artificial chromosome (PAC) harboring the gene expression regulatory region have advantages, albeit the moderate expression level of transgene. In addition, because few models exhibit DA neuronal loss and associated motor phenotype, the progression of prodromal to motor phase is difficult to be assessed. Nonetheless, these models, even without DA cell loss, can be useful for the development of symptomatic and disease-modifying therapies targeting α-syn-related non-motor symptoms.

##### Prion Promoter

The transgenic mice overexpressing A53T α-syn under the prion promoter were first reported in 2002. They showed robust α-syn aggregation, especially in the spinal cord, leading to motor paralysis and death. As for non-motor symptoms, it is reported that they showed a deficit of odor discrimination and odor detection at 6 months [59]. In this report, the levels of phosphorylated α-syn and TH in the olfactory bulb were increased, and cholinergic denervation of the mitral cell layer was observed before the onset of motor symptoms. Another report showed that the GI motility was attenuated at 3 months, and α-syn aggregates were observed in the mesenteric and submucosal plexus of the colon [60]. These GI phenotypes were already observed when mice displayed only faint central nervous pathology without motor symptoms, suggesting that this could be a viable model for premotor GI dysfunction. 

##### Thy1 Promoter

Transgenic mice overexpressing wild-type α-syn under the Thy1 promotor showed hyposmia and insoluble α-syn inclusion in the olfactory bulb at 3 to 5 months [61]. Anxiety appears at 4 months, sleep disturbance occurs at 9–10 months, and colonic dysfunction are seen at 12 months. Although the number of TH neurons in the SNc did not decrease even at 22 months, the locomotion became reduced with the decrease of striatal DA content by 40% after 14 months, and it is considered to be attributed to the DA terminal loss [62]. This model is especially useful to test the therapeutics to slow the progression of the disease in a prodromal phase to the motor phase, and to test the effect of intervention to prodromal symptoms on the development of motor symptoms. 

##### Endogenous Promoter Using BAC or PAC

Transgenic human A53T α-syn mice with P1-derived artificial chromosome (PAC) on an endogenous α-syn null background showed reduced intestinal motility and prolonged whole-gut transit time at 3 months along with a decreased stool water content. Pathologically, α-syn was accumulated in the intestinal submucosa and myenteric plexus [63]. They do not show olfactory loss but show motor symptoms at 6 months. However, there are no significant TH neuronal loss in SNc and decrease of the content of DA, norepinephrine, or serotonin in the striatum by HPLC. In another report, wild-type human *SNCA* BAC transgenic mice showed no significant change in the content of DA, norepinephrine, or serotonin by HPLC but exhibited increased dopamine and serotonin transporter expression [22].Wild-type human *SNCA* BAC transgenic mice on an endogenous α-syn null background exhibited a constipation-like phenotype that was only seen in male mice before motor symptoms [64]. We recently reported A53T α-syn BAC transgenic mice showing an RBD-like phenotype and hyposmia [65]. These mice exhibited REM sleep without atonia, which is a key feature of RBD, at as early as 5 months of age, and olfactory dysfunction by 9 months. In these mice, phosphorylated α-syn was found to be accumulated in the dorsomedial medulla and sublaterodorsal nucleus, as well as the olfactory bulb, and the number of TH-positive neurons in the SNc was decreased by 18% without motor symptoms at 18 months. This phenotype suggests that this is a prodromal PD mouse model. Sleep abnormalities, most of which involve changes in the architecture of sleep, have been reported in a number of animal models, so a comprehensive review of the rodent model would be useful [66]. However, there have been no α-syn-based genetic animal models reported that exhibit RBD with the exception of the abovementioned A53T α-syn BAC transgenic mice. 

In a rat model, wild-type α-syn BAC transgenic rats showed olfactory dysfunction at 3 months [67]. They showed a decrease in the content of DA in striatum by 30% at 6 months, followed by the motor phenotype at 16 months. They also showed anxiety-like behaviors, but there are no significant changes in the contents of noradrenaline and serotonin. 

#### 2.3.2. Other Genetic Models

Genetic mutations that lead to the formation of Lewy bodies are important for creating the animal model of idiopathic PD. It has been reported that LRRK2 R1441C knock-in mice showed reduced anxiety-like behaviors at about 8 to 10 months, and motor and olfactory dysfunction at 24 to 26 months [68]. In LRRK2 R1441G BAC transgenic mice, gastrointestinal dysfunctions began at 6 months, followed by mild hypokinesia in the open field at 16 months, but other non-motor phenotypes were not observed [69].

### 2.4. Prodromal Symptoms of α-syn Propagation Models

A lot of α-syn fibril injection models are reported, but models with injection into the olfactory bulb and GI tract are especially important to trace the propagation of α-syn pathology and related non-motor symptoms as well as to validate Braak’s hypothesis. 

#### 2.4.1. Olfactory Injection

According to a study in which α-syn fibrils were injected into the olfactory bulb of mice, olfactory disturbance appeared after 1 month [70,71]. In these studies, phosphorylated α-syn first appeared in the olfactory bulb, spread to the hippocampus and amygdala 3 months later, and was transmitted to the SNc and locus coeruleus 12 months after the injection. However, they showed neither cognitive decline, psychiatric symptoms, nor motor dysfunction. 

#### 2.4.2. GI Tract Injection

Several α-syn injection models into the GI tract have been reported. The initially reported mice model injected with α-syn fibrils into the stomach showed the transient formation of α-syn aggregates in the DMV without further upward propagation [31,32]. Similarly, injections of α-syn fibrils into the colon of rats and non-human primates (cynomolgus monkeys) have only resulted in faint brainstem lesions and transient GI symptoms [72]. However, mice injected with α-syn fibrils into the stomach and duodenum exhibited propagation of α-syn pathology from the brainstem to the midbrain, amygdala, hippocampus, and eventually to the olfactory bulb [33]. In accordance with this pathological progression, the stool number per passage and water content were decreased at 1 month post-inoculation (p.i.); motor symptoms, anxiety/depression, and memory impairment appeared at 7 months p.i.; finally, olfactory impairment appeared at 9 months p.i. in these mice. This important propagation model partly reproduced the natural history of PD from the prodromal to symptomatic stages. However, various factors, such as the method of fibril preparation, the site of injection, and the amount of injected α-syn fibrils, seem to have effects on the degree of α-syn propagation so further examinations will be necessary.

As a novel delivery method of α-syn fibrils, intravenous administration using rabies virus-derived neuron-targeting peptide (RVG9R) has been reported [73]. In this model, non-motor symptoms, such as diminished intestinal motility and olfactory disturbance, were recognized 6 months after the administration of α-syn fibrils, and the accumulation of phosphorylated α-syn was observed in the myenteric plexus of the duodenum. In the central nervous system, phosphorylated α-syn began to accumulate in the DMV after 4 months, and the α-syn pathology spreads rostrally to the locus coeruleus, the substantia nigra, and the cortex. However, the number of TH cells in the SNc was decreased by only 15%-20%. Although the distribution of these lesions may depend on the cell-type preference of RVG9R as well as the expression of endogenous α-syn, it attracts attention as a novel prodromal PD model.

## 3. Conclusions

A number of animal models have helped clarify the pathomechanisms underlying PD, but none has faithfully reproduced the entire natural history of PD. An ideal model of prodromal PD is one that reproduces several PD-specific premotor symptoms followed by the slowly progressive DA neurodegeneration. From this viewpoint, mice with α-syn fibrils injected into the GI tract that show hyposmia, GI symptoms, and DA cell loss with robust α-syn propagation seem to be a promising model for prodromal PD, despite the artificial element of α-syn fibril inoculation. [33]. A53T *SNCA* BAC transgenic mice also show both hyposmia and RBD symptoms with phosphorylated α-syn accumulation and mild DA cell death in late stages [65]. This model partly reproduces the initiation process of PD in that multiple non-motor symptoms, including hyposmia and RBD, are observed before the onset of motor symptoms, which is difficult to fully explain by the α-syn propagation alone.

It is difficult to reproduce all the aspects of the preclinical, prodromal, and advanced stages of PD in a single animal model, so models should be selected according to the experimental purpose. If genetic factors are weighted, α-syn overexpression and the multiple initiation model are valued, and if environmental factors are weighted, the α-syn propagation model is useful. In reality, the propagation from the multiple initiation sites may be occurring in PD. α-Syn fibril injection to the GI tract and olfactory bulb to human α-syn transgenic mice will be useful to trace the progression of α-syn pathology and to test the therapeutics against the progression of human α-syn toxicity in vivo.

Animal models continue to play an important role in the field of PD research, and the prodromal PD model will provide a substantial contribution to research efforts, especially the development of DMT and early biomarkers for PD in the future.

## Figures and Tables

**Table 1:** Advantages and disadvantages of each model.

	Toxin-Based Model	α-syn Transgenic Model	α-syn Propagation Model
**Phenotype** **(Motor)**	Robust	Rare	Possible (requires reproducibility)
**Phenotype** **(Non-Motor)**	Rare	Widespread	Possible (requires reproducibility)
**Time Required for Experiments**	Short (days~weeks)	Long (months~years)	Moderate (1~12 months)
**Animal Species**	Applicable to non-human primates	Difficult to apply to non-human primates	Applicable to non-human primates
**Precautions in Experiments** **and Interpretations**	Risk of exposure to toxins	Off-target effect of transgene	Risk of exposure to fibrils (prionoids) Various protocols in relation to fibrils
**Comparison with Etiology in Human**	Different from etiology in human	Close to etiology in human	Reproduces a part of etiology in human
